# Impact of a Translated Disease Self-Management Program on Employee Health and Productivity: Six-Month Findings from a Randomized Controlled Trial

**DOI:** 10.3390/ijerph15050851

**Published:** 2018-04-25

**Authors:** Matthew Lee Smith, Mark G. Wilson, Melissa M. Robertson, Heather M. Padilla, Heather Zuercher, Robert Vandenberg, Phaedra Corso, Kate Lorig, Diana D. Laurent, David M. DeJoy

**Affiliations:** 1 Center for Population Health and Aging, Texas A&M University, College Station, TX 77843, USA; 2 Department of Environmental and Occupational Health, School of Public Health, Texas A&M University, College Station, TX 77843, USA; 3 Workplace Health Group, Department of Health Promotion and Behavior, College of Public Health, The University of Georgia, Athens, GA 30602, USA; mwilson@uga.edu (M.G.W.); hmbowen@uga.edu (H.M.P.); zuercher@uga.edu (H.Z.); dmdejoy@uga.edu (D.M.D.); 4 Department of Psychology, The University of Georgia, Athens, GA 30602, USA; robertsonmelissa27@gmail.com; 5 Department of Management, Terry College of Business, The University of Georgia, Athens, GA 30602, USA; rvandenb@uga.edu; 6 Department of Health Policy and Management, College of Public Health, The University of Georgia, Athens, GA 30602, USA; pcorso@uga.edu; 7 Self-Management Resource Center, Palo Alto, CA 94303, USA; kate@selfmanagementresource.com (K.L.); diana@selfmanagementresource.com (D.D.L.)

**Keywords:** disease self-management, employee health, intervention translation, evidence-based program, Chronic Disease Self-Management Program, United States of America

## Abstract

Disease management is gaining importance in workplace health promotion given the aging workforce and rising chronic disease prevalence. The Chronic Disease Self-Management Program (CDSMP) is an effective intervention widely offered in diverse community settings; however, adoption remains low in workplace settings. As part of a larger NIH-funded randomized controlled trial, this study examines the effectiveness of a worksite-tailored version of CDSMP (wCDSMP [*n* = 72]) relative to CDSMP (‘Usual Care’ [*n* = 109]) to improve health and work performance among employees with one or more chronic conditions. Multiple-group latent-difference score models with sandwich estimators were fitted to identify changes from baseline to 6-month follow-up. Overall, participants were primarily female (87%), non-Hispanic white (62%), and obese (73%). On average, participants were age 48 (range: 23–72) and self-reported 3.25 chronic conditions (range: 1–16). The most commonly reported conditions were high cholesterol (45%), high blood pressure (45%), anxiety/emotional/mental health condition (26%), and diabetes (25%). Among wCDSMP participants, significant improvements were observed for physically unhealthy days (uΔ = −2.07, *p* = 0.018), fatigue (uΔ = −2.88, *p* = 0.002), sedentary behavior (uΔ = −4.49, *p* = 0.018), soda/sugar beverage consumption (uΔ = −0.78, *p* = 0.028), and fast food intake (uΔ = −0.76, *p* = 0.009) from baseline to follow-up. Significant improvements in patient–provider communication (uΔ = 0.46, *p* = 0.031) and mental work limitations (uΔ = −8.89, *p* = 0.010) were also observed from baseline to follow-up. Relative to Usual Care, wCDSMP participants reported significantly larger improvements in fatigue, physical activity, soda/sugar beverage consumption, and mental work limitations (*p* < 0.05). The translation of Usual Care (content and format) has potential to improve health among employees with chronic conditions and increase uptake in workplace settings.

## 1. Introduction

Globally, organizations understand the importance of a healthy, engaged, and productive workforce. In 2016, there were over 159 million employed adults in the United States (U.S.) alone, and 48% were between the ages of 40 to 64 years [1]. Between 22% and 49% of employees are estimated to have one or more chronic conditions [2]. These conditions and associated symptoms can cause working adults to experience problems meeting work demands [2] and eventually lead to disability and/or unplanned or premature workforce departure [3,4,5]. 

Workplace health promotion initiatives have the potential to improve employee health, increase worker productivity, and decrease healthcare costs [6,7,8,9]. In a multi-employer study examining direct and indirect healthcare-related costs and self-reported productivity among employees, obesity, arthritis, back pain, and depression were identified being among the top five most expensive health conditions [10]. Evidence suggests that offering disease self-management interventions to employees can result in substantial benefits to employees and employers [8]. While the rising prevalence of chronic conditions among working-age adults reinforces the need to increase disease self-management interventions, less is known about the best strategies to deliver and diffuse effective evidence-based disease management programs to employees in workplace settings. 

The Chronic Disease Self-Management Program (CDSMP) is among the most widely disseminated and evaluated evidence-based program for middle-aged and older adults in the United States [11,12]. CDSMP is a process-based intervention based on Social Learning Theory [13] and is appropriate for any adult living with one or more chronic conditions (e.g., cardiovascular disease, diabetes, depression, cancer). The intervention is most commonly offered in small-group workshops with approximately 8 to 18 participants [14]. The workshops are led by two trained facilitators and consist of six 2.5-h sessions, which are delivered once per week for six consecutive weeks. During the workshops, problem solving, action planning, and goal setting are used to build participants skills to manage their disease symptomology. Examples of topics discussed during the workshops include techniques to cope with health problems and negative emotions, nutrition, physical activity, how to assess new treatments, and communication with healthcare providers. CDSMP was deemed efficacious in the late 1990s through a randomized controlled trial (RCT) [15,16,17]. From that time, the program has been translated for community use and disseminated through a national trainings, certification, and licensure infrastructure [18]. Among the robust findings from this grand-scale dissemination, the National Study of CDSMP documented the intervention’s maintained effectiveness in terms of activity limitations, depression, and quality of life [19,20]. Based on its long history of effectiveness, CDSMP has been translated to tailor the intervention for specific conditions (e.g., diabetes, arthritis, chronic pain, cancer) and languages (i.e., primarily Spanish, but available in approximately 17 languages). 

Historically, CDSMP has been primarily delivered through the aging services network, with predominant delivery sites being senior centers, healthcare organizations, residential facilities, and faith-based organizations [12,21]. An examination of delivery sites for the national rollout of CDSMP through the Administration for Community Living reveals precisely how low the uptake has been in workplace settings [21]. Of the 201,587 participants enrolled in CDSMP spanning 47 states from December 2009 to December 2016, only 2243 (i.e., 1.1%) attended workshops in workplace settings. 

The current study presents preliminary findings from a RCT to translate CDSMP for use in the workplace. Data from this study were used to examine changes from baseline to 6-month follow-up as part of a larger, ongoing trial. Justification for this translation was based on recognition that (1) the United States workforce is aging; (2) working-aged adults are being diagnosed with chronic conditions at younger ages; (3) effective disease self-management interventions are needed in workplace settings; and (4) the existing CDSMP format causes logistical issues that hinder its delivery in workplace settings [22]. This study assesses the translation of CDSMP for use in the workplace. More specifically, this study examines the effectiveness of a worksite-tailored version of CDSMP (i.e., wCDSMP) relative to CDSMP (i.e., ‘Usual Care’) to improve health and work performance among employees with one or more chronic conditions.

## 2. Materials and Methods

### 2.1. Translation Process

This study was funded by the National Heart, Lung, and Blood Institute to translate CDSMP for use in the workplace among employees. In collaboration with the original program developer, the first year of this 5-year grant was dedicated to modifying the intervention’s format and content to be more appropriate for on-site workplace delivery while meeting the needs of younger employed participants. The program translation was guided by the research team’s experience translating interventions to worksite settings [23,24] and implementing and evaluating CDSMP in community and healthcare settings [11,19,21]. To inform the translation, the research team gathered input from CDSMP Master Trainers (i.e., national survey, interviews) and employees who had one or more chronic diseases (i.e., focus groups). Master Trainers were selected as participants because of the extensive training they received about the intervention (e.g., content, processes, fidelity), their role hosting lay leader trainings for those who facilitate workshops, and their experience interacting with adults living with chronic conditions. Employees with chronic diseases were selected as participants because of their first-hand experiences living and working with a chronic condition as well as their experience participating in health promotion activities at work. Additionally, the research team engaged in extensive conversations with the program developers throughout the translation process regarding program logistics (e.g., grand-scale dissemination, translation, leader training, fidelity monitoring). Information obtained from these sources was used to develop materials for the translated intervention. The translation resulted in a worksite-tailored version of CDSMP that is referred to as wCDSMP (also known in the community as Live Healthy, Work Healthy).

Unique aspects of wCDSMP relative to CDSMP are provided in Table 1. In the formative research described above, Master Trainers indicated that the length (2.5 h) of CDSMP workshop sessions was the most common barrier to implementing workshops in worksite settings. As such, the wCDSMP workshop format was revised to include sixteen 50-min sessions that occur twice a week for eight weeks. When delivered to employees at a worksite, both interventions can be delivered on- or off-site or during or outside of work time (at the discretion of the employer). Both interventions require that workshops be facilitated by two leaders who have completed the 4-day standardized training. To facilitate wCDSMP, leaders must attend an additional bridge training (approximately 4 h). The primary target population for wCDSMP is adults aged 40 years and older with one or more chronic condition, which is younger than the traditionally targeted CDSMP population [25]. As with CDSMP, wCDSMP workshops aim to enroll a maximum of 16 participants to ensure adequate time is allotted for feedback and action planning activities.

The two interventions share much of the same content (approximately 75%). To accommodate the twice weekly sessions in wCDSMP, the order of activities was modified. Generally, wCDSMP has included an emphasis on work-life balance and contains additional work-related/focused examples, content, and activities. Revisions were made to include additional activities addressing stress management and refine the communication activity to transcend healthcare providers to include co-workers and supervisors. Information related to nutrition was updated and streamlined (relying more on the book), and content related to falls was omitted. 

### 2.2. Recruitment

This RCT was implemented in two rural communities in South Georgia. The local YMCAs were selected as the primary community contact based on their existing organizational relationships/partnerships and experience delivering group-based programs [26]. Responsibilities of the YMCA for this study were to have staff trained as Master Trainers, host lay leader trainings, identify potential workplaces as study sites, assist in organization and participant recruitment, facilitate workshops, and assist in data collection.

Working with the YMCA staff, the research team met with leadership at potential worksites to introduce them to the intervention and recruit them to participate in the study. Sites were recruited in stages to ensure the volume of participants could be enrolled in workshops based on the newly formed delivery infrastructure. The research team conducted interviews with worksite leadership to learn more about each organization, employee characteristics, and past success with workplace health programs as well as to formulate strategies to recruit participants and deliver the workshops.

This study includes data from nine organizations enrolled as intervention sites. Sites were randomized at the organization-level to avoid cross-contamination across intervention arms. To assist in the randomization process, organizations were collapsed or separated based on employee size. Sites randomized to receive wCDSMP included a regional medical center, two county school systems (i.e., middle school, junior high school, high school, central office), a community action agency, and a processing plant. Sites randomized to receive CDSMP included the city government, county government, a behavioral healthcare facility, a bank, two county school systems (i.e., elementary schools), and a technical college. Intervention workshops were made available to all full-employees at each site. Workshop participants included a mix of employee types with differing roles and supervisory responsibilities. Participants may have known one another prior to workshop participation, and many workshops included participants from multiple organizational divisions (where applicable). Employee participation in workshops was voluntary. There were no eligibility criteria for employees to participate in intervention workshops; however, recruitment efforts aimed to enroll employees with one or more chronic condition. Strategies used to recruit participants were primarily dictated by site-specific recommendations and typically included a combination of emails, brief presentations during regularly scheduled meetings, flyers on bulletin boards, and on-site registration (research team members being physically present in break rooms or other site venues). Sessions were scheduled at times that fit the organization’s work schedule. A total of 10 wCDSMP workshops and 13 CDSMP workshops were delivered between January 2016 and February 2017, 10 of which were after work hours (all at schools), and 13 of which were during work hours (all other locations).

### 2.3. Data Collection

For this study, participants completed an instrument at baseline (i.e., prior to the first workshop session) and 6-month follow-up. The instruments asked participants to provide information related to their health status, perceptions of work performance, healthcare utilization, and sociodemographics. Most participants completed the instrument online, although paper-based surveys were available onsite upon request. A clinical partner in this RCT collected a fasting blood draw that was analyzed for glucose, cholesterol, and high-sensitivity C-reactive protein at baseline and 6-month follow-up. Participant height, weight, and blood pressure were measured by trained research staff. An incentive in the form of a US$10 gift card was provided to participants at each data collection. The University of Georgia Institutional Review Board (study #MOD00005416) approved all study materials and procedures.

### 2.4. Outcome Measures

Unhealthy Days. Two items from the CDC Healthy Days Scale were used to assess participants’ self-reported unhealthy days [27]. Participants were asked, “Thinking about your physical health, which includes physical illness and injury, for how many days during the past 30 days was your physical health not good?” Participants’ responses could range from 0 to 30 days [28]. Participants were then asked, “Thinking about your mental health, which includes stress, depression, and problems with emotions, for how many days during the past 30 days was your mental health not good?” Again, participants’ responses could range from 0 to 30 days. These variables were treated continuously in analyses.

Single-Item Health Indicators. Self-reported levels of stress, pain, fatigue, and sleep problems were each measured using 11-point Likert-type items [27,29]. Participants were asked to indicate the degree to which they experienced these issues in the past week using scales ranging from 0 (none) to 10 (severe). These variables were assessed separately and treated continuously in analyses.

Depression. The patient health questionnaire (PHQ)-8 was used to assess participants’ depression symptomology [30]. Participants were asked to respond to eight items based on the following stem: “Over the last 2 weeks, how often have you been bothered by any of the following problems?” Example items included, little interest or pleasure in doing things, “feeling down, depressed, or hopeless”, and “feeling bad about yourself—or that you are a failure or have let yourself or your family down”. Each item was scored using a 4-point Likert-type scale with response categories of “not at all”, “several days”, “more than half the days”, and “nearly every day”. A composite scale was calculated for each participant by summing their responses. Possible scores ranged from 0 to 24. This variable was treated continuously in analyses, with higher scores indicating more depressive symptomology.

Eating Behavior. Participants’ eating behaviors over the past week were measured using three items [31]. Participants were asked, “Over the past seven days, how many times did you eat fast food meals or snacks?” Response choices ranged from 0 to 5+ times. Participants were asked, “Over the past seven days, how many servings of fruits/vegetables did you eat each day?” Response choices ranged from 0 to 5+ servings. Participants were asked, “Over the past seven days, how many soda or sugar-sweetened drinks (regular, not diet) did you drink each day?” Response choices ranged from 0 to 5+ drinks. 

Physical Activity and Sedentary Behavior. Participants’ physical activity was measured in two ways [29]. First, participants were asked, “How many days in the past week were you physically active or exercising for at least 30 min, such as brisk walking, running, dancing, bicycling, water exercise, etc., that may cause faster breathing or heartbeat, or feeling warmer (it does not have to be all at one time)?” Response choices ranged from 0 to 7 days in the past week. Then, participants were asked to estimate the total hours they spent sitting during a typical work day using a series of four items (i.e., while traveling to or from places, as part of their job, while watching TV/using a computer not at work, for recreation) [32]. The total number of reported hours were summed and treated as a continuous variable in analyses. 

Self-Efficacy. Participants’ self-efficacy to manage their chronic conditions was measured using a 7-item scale [29]. Examples of items within the scale include, “How confident are you that you can keep any other symptoms or health problems you have from interfering with the things you want to do?” and “How confident are you that you can do the different tasks and activities needed to manage your health condition so as to reduce your need to see a doctor?” Items were scored on a scale from 0 (not at all confident) to 10 (completely confident). Responses for these items were averaged to create a composite variable where higher scores indicate higher self-efficacy to manage conditions.

Medication Adherence. Participants were asked to report adherence to their prescribed medications using a 4-item scale [33]. Examples of items within the scale include, “Do you ever forget to take your medication?” and “When you feel better, do you sometimes stop taking your medicine?” Response categories for each item were “no” and “yes”. Items were summed to create a composite score where higher scores indicate worse prescribed medication adherence.

Patient–Provider Communication. Participants were asked to report aspects of their communication with physicians using a 3-item scale [29]. Examples of items within the scale include, “How often do you prepare a list of questions for your doctor?” and “How often do you ask questions about the things that you want to know and things you don’t understand about your treatment?” Response categories ranged from 1 (never) to 6 (always). Responses for these items were averaged to create a composite score where higher scores indicate better communication with physicians.

Work-Related Limitations. The short-form Work Limitations Questionnaire was used to assess participants’ work-related demands in terms of time (mean of 2 items), mental (1 item), interpersonal (1 item), and output (mean of 2 items) domains [34]. Each item was scored using a 5-point scale and asked participants to report work-related limitations/difficulties over the past two-week period. Response choices for these items ranged from 0 (difficult none of the time [0%]) to 4 (difficult all of the time [100%]). Scores on each subscale were multiplied by 25 so that each score represents the percentage of work time affected by physical health or emotional problems. Each of the four domains were analyzed separately in analyses, with higher scores indicating more limitation/difficulty.

Work Ability. Self-reported work ability was measured using a modified version of the Work Ability Index [35]. Scores on this measure were derived from a number of items reflecting participants’ chronic health conditions, self-reported ability to work, well-being over the past three months, and description of job demands. This variable was treated continuously in analyses, with higher scores representing greater ability to work.

Work-Related Stress. Participants were asked to report their job stress using three randomly selected items from Cohen et al. (1983) adapted to measure stress at work [36]. Participants were asked, “In the last month, how often have you felt that you were unable to control the important things at work?” and “In the past month, how often have you found that you could not cope with all the things you had to do at work?” Response categories ranged from 0 (never) to 4 (very often). Responses for these items were averaged to create a composite measure; higher scores indicate more work-related stress. 

### 2.5. Statistical Methods

Descriptive statistics were performed on participant characteristics collected baseline, which were compared by intervention condition. Chi-square tests were used to identify distribution differences for categorical variables, and independent-sample tests were used to identify mean differences for count and continuous variables (see Table 2). These analyses do not account for non-independence of observations due to shared worksites. To test differences between the two conditions in outcomes, multiple-group latent-difference score models [37,38] were fitted to examine changes from baseline to 6-month follow-up for outcomes of interest. These models use path constraints to create new latent (i.e., unobserved) variables, which represent the change in the variable between baseline and follow-up. The relevant model parameters involve the mean of the changes (uΔ) and the variance of the changes (σ2Δ). MPlus 7.2 [39] was used to test these multiple-group latent-difference score models. A sandwich estimator (i.e., the MPlus CLUSTER command) was included to adjust standard errors for non-independence due to the inclusion of multiple worksites within each intervention condition.

**Figure 1:**
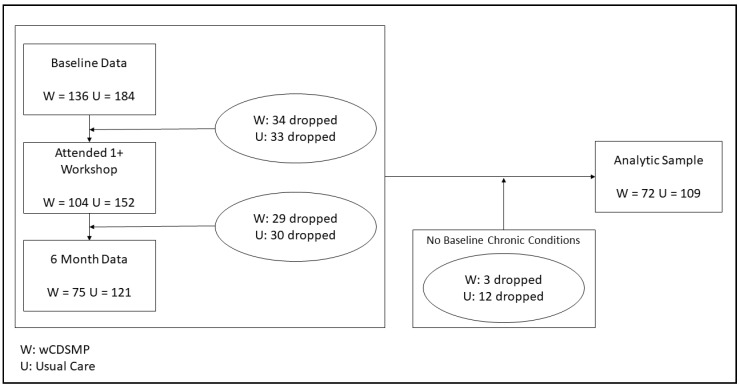
CONSORT diagram depicting participant flow.

Models for each outcome variable were fitted separately for both intervention conditions (i.e., CDSMP and wCDSMP). All models controlled for proportion of workshop sessions attended by regressing the latent change scores on this variable. Analyses also accounted for baseline levels of the dependent variable by covarying baseline levels with latent change scores. Effect sizes were derived by dividing the latent difference score by the standard deviation of the changes (see Shubert et al. for a similar strategy [40]). Between-group differences in latent change score means were tested by subtracting the CDSMP mean change from the wCDSMP mean change using the MPlus MODEL CONSTRAINT feature; these analyses controlled for age, proportion of sessions attended, and number of chronic conditions at baseline. Parameters were estimated using a robust maximum likelihood estimator. As seen in Figure 1, participants without data at both time points, those who did not attend 1+ workshop sessions, and those who did not self-report one or more chronic condition(s) were omitted from study analyses. The final analytic sample contained 181 participants (*n* = 109 CDSMP participants and 72 wCDSMP participants).

## 3. Results

Table 2 presents sample characteristics by condition. The majority of participants were female (87.1%) and non-Hispanic white (62.2%). Over 26% of participants had some college or technical/vocational training, 14.3% had an associate’s degree, and 34.9% had a graduate degree. On average, participants were 47.90 (±10.10) years of age, had a body mass index of 34.90 (±7.91), and self-reported 3.25 (±2.02) chronic conditions. The most frequently reported conditions were obesity (73.3%), high cholesterol (45.1%), high blood pressure (44.8%), anxiety or other emotional/mental health condition (26.4%), diabetes (25.1%), musculoskeletal injury/disorder (22.7%), depression (22.1%), and arthritis or other rheumatic disease (19.9%). At baseline, 41.6% of participants screened had elevated total cholesterol, 33.9% had elevated glucose, 30.3% had elevated diastolic blood pressure, and 23.6% had elevated systolic blood pressure. On average, participants attended 61% of the workshop sessions offered.

When comparing participant characteristics by condition, wCDSMP participants were significantly older than CDSMP participants (49.99 years compared to 46.51 years). On average, wCDSMP participants reported significantly more chronic conditions (3.64 conditions compared to 3.00 conditions). A significantly larger proportion of wCDSMP participants self-reported having high blood pressure (54.2% compared to 38.5%) and diabetes (37.1% compared to 17.1%) relative to CDSMP participants. A larger proportion of wCDSMP participants also had elevated glucose levels (43.7% compared to 27.4%). On average, wCDSMP participants attended a significantly smaller proportion of workshop sessions (52% compared to 68%).

Table 3 presents results of latent change regression analyses by condition from baseline to 6-month follow-up. All analyses controlled for proportion of total workshop sessions attended to control for intervention dose. Among wCDSMP participants only, significant reductions in physically unhealthy days (uΔ = −2.07, S.E. = 0.87, *p* = 0.018, E.S. = −0.02) and fatigue (uΔ = −2.88, S.E. = 0.92, *p* = 0.002, E.S. = −0.17) were observed from baseline to follow-up. Significant reductions in soda/sugar beverage consumption (uΔ = −0.78, S.E. = 0.35, *p* = 0.028, E.S. = −0.24), fast food intake (uΔ = −0.76, S.E. = 0.29, *p* = 0.009, E.S. = −0.27), and sedentary behavior (uΔ = −4.49, S.E. = 1.90, *p* = 0.018, E.S. = −0.02) were observed from baseline to follow-up. Significant improvement in patient–provider communication was observed (uΔ = 0.46, S.E. = 0.21, *p* = 0.031, E.S. = 0.33). Significant work limitation reductions in terms of mental demands (uΔ = −8.89, S.E. = 4.47, *p* = 0.010, E.S. = −0.02) were also observed from baseline to follow-up. 

As shown in Table 2, wCDSMP and CDSMP differed in terms of proportion of sessions attended, number of chronic conditions, and age. To account for these differences in estimating between-group differences in change, we regressed the change scores on these three covariates (see Table 3, Between-Group). When comparing intervention effectiveness based on between-group differences in latent change scores, improvements in fatigue (uΔ = −3.68, S.E. = 1.31, *p* = 0.005), soda/sugar beverage consumption (uΔ = −2.70, S.E. = 0.92, *p* = 0.003), and physical activity (uΔ = 2.88, S.E. = 1.40, *p* = 0.039) were significantly greater among wCDSMP participants relative to Usual Care participants. Relative to Usual Care participants, work limitation reductions were significantly greater among wCDSMP participants for mental demands (uΔ = −30.56, S.E. = 14.87, *p* = 0.040). 

## 4. Discussion

Findings from this RCT show the effectiveness of a workplace-tailored version of CDSMP (i.e., wCDSMP) to improve health and work performance indicators among employed adults with one or more chronic conditions. Consistent with previously reported results from the National Study of CDSMP, wCDSMP was associated with reductions in unhealthy physical days, fatigue, physical activity, and patient–provider communication [19,20]. These results are consistent with previous age-based comparisons demonstrating that middle-aged CDSMP participants reported stronger improvements in these outcomes relative to their older adult counterparts [41]. Given the average age of wCDSMP participants was approximately 17 years younger than those in National Study of CDSMP (i.e., 50.0 years compared to 67.0 years), the current study supports the benefits of disease self-management skills for improving health and indicates that the worksite translation of CDSMP is associated with health benefits among a younger employed population living with chronic conditions. Compared to many older adults who are no longer working, middle-aged employees with chronic conditions are often challenged to simultaneously manage their health, work responsibilities, and home/family life [5,42,43]. As such, their health needs and strategies for self-management may differ from their older counterparts. As part of the wCDSMP translation process [43,44], program content was modified to emphasize the importance of work-life balance. Additionally, content was modified to include more age-appropriate, work-related examples and include work-focused brainstorming and problem solving activities. Consequently, this translational study included novel work-related outcomes that have not previously been included in other CDSMP studies. Specifically, wCDSMP participants reported decreased difficulty performing mental demands at work and reduced sedentary activity on workdays. These findings demonstrate that wCDSMP is an effective translation capable of yielding important, previously understudied outcomes and benefits for a younger, employed population. Further, this translation indicates that traditional CDSMP may not be a good fit for working adults, evidenced by non-significant outcomes among Usual Care participants (despite having higher attendance than wCDSMP participants). 

Given over 80% of wCDSMP participants were obese (compared to 68.5% of CDSMP participants), it is not surprising that the tailored intervention improved physical health, fatigue, eating behavior, and sedentary behavior on workdays. However, it is surprising that improvements in medication adherence and self-efficacy did not significantly improve in light of the large proportion of participants with elevated biomarkers at baseline (e.g., glucose, blood pressure, cholesterol). However, this may have resulted from relatively high disease management self-efficacy and medication adherence at baseline (see Table 1). In future analyses, changes in biomarkers will be examined, especially in the context of mediating and moderating variables that may reveal improvements for employee subgroups.

As indicated by its low national uptake [21], the traditional format of CDSMP was not conducive to the adoption and delivery in workplace settings [22]. The 2.5-h workshop sessions reduced employers’ ability or willingness to implement the intervention at the workplace, especially on work time [24]. As part of the wCDSMP translation, workshop sessions were shortened and held more frequently, which may facilitate increased acceptability by management in workplace settings. While wCDSMP attendance was lower than preferred (i.e., on average, participants attended 52% of workshop sessions), some intervention benefits were nevertheless observed from baseline to 6-month follow-up. Additional efforts are needed to examine minimal attendance threshold levels needed for programmatic success on different outcome variables, and increase participant retention accordingly. 

It is unclear whether low attendance was associated with unpredictable job-related responsibilities/emergencies/deadlines, lack of interest, competing personal and family responsibilities, or changes in work schedules (e.g., rotating shift work) due to the nature of the worksites included in this study (i.e., hospital and school employees). While the modified format of wCDSMP may be more feasible for on-site delivery (i.e., before, during, and/or after work time), the length of the intervention (i.e., sixteen 50-min sessions over eight weeks) may hinder grand scale adoption. To improve worksite adoption/uptake and increase intervention dose received by employees, the intervention should be further refined to include fewer sessions (e.g., twelve 60-min sessions over six weeks). Possible strategies for increasing workshop attendance may include the inclusion of a Session Zero [45] for enhanced intervention recruitment or creating toolkits and trainings to educate employers and program deliverers (e.g., local Area Agencies on Aging) about effective recruitment and implementation strategies. Additionally, efforts are needed to identify the interests and preferences of employees with chronic conditions in terms of intervention (types and format) delivered in workplace settings [46,47]. 

### Limitations

This study was not without limitations. Data were self-reported, which may have introduced recall and reporting biases. While this study focused on many aspects of health and work performance, additional outcome measures should be examined to capture the breadth of possible intervention benefits. Future studies from this RCT will investigate a broader range of outcomes related to health and healthcare utilization to further confirm the success of this translational research. Further, additional measures associated with work performance will be examined to assess the intervention’s cost-effectiveness and return on investment. While wCDSMP participants reported improvements for all outcome variables, the sample size for this RCT was modest and may have been underpowered to detect all meaningful changes. Further, all participants reporting one or more chronic conditions were included in analyses, not just those who reported risk or poor health indicators at baseline. Future analyses will examine intervention benefits for each outcome variable in terms of those who reported associated risk for this outcome at baseline (e.g., stress, depression, sleep problems). In our ongoing RCT, we are working to increase the study sample to include more employees at more worksites (and more diverse worksite types) for a more comprehensive understanding of impacts in different populations and settings. This will also help us better understand the appropriateness of wCDSMP and its effectiveness for certain types of work environments and employee job descriptions. The current study examined changes over a 6-month period; however, our RCT will examine changes over a 12-month period to determine the ability of wCDSMP to maintain employee health and productivity over time, relative to Usual Care alternatives.

## 5. Conclusions

Despite increased awareness about the importance of workplace health promotion, small proportions of employed Americans report that their employers offer resources to manage their health needs [48]. Furthermore, even fewer programs have undergone rigorous development and testing as an evidence-based worksite wellness program. Given the well-documented effectiveness of CDSMP, efforts are needed to increase adoption and dissemination to reach new populations and settings. This study showed the effectiveness of wCDSMP to improve health and work performance among middle-aged employees with one or more chronic conditions. By translating the intervention format and content, we believe wCDSMP can increase the availability and accessibility of this efficacious intervention to workplace settings. The added flexibility afforded by the wCDSMP format has potential to increase its dissemination workplace settings across the U.S. and globally. Employers who offer wCDSMP at their workplace can promote healthy aging among their mid-aged workforce and prevent or delay costly age- and disease-related complications and disability. 

## Figures and Tables

**Table 1:** Comparison of the Chronic Disease Self-Management Program (CDSMP) and the worksite-tailored version of CDSMP (wCDSMP) workshop delivery.

CDSMP (Usual Care)	wCDSMP (Workplace-Tailored)
Format	
**6** weeks	**8** weeks
**2.5** h sessions (**1** session per week)	**50** min sessions (**2** sessions per week)
On-site or off-site (worksite dependent)	On-site or off-site (worksite dependent)
On or off work time (worksite dependent)	On or off work time (worksite dependent)
Facilitated by 2 leaders	Facilitated by 2 leaders
Leader training (4-day training)	Leader Training (4-day training) + **bridge training (4 h)**
Participant materials (book & CD)	Participant materials (book & CD) [consider lending library]
Target participants aged 50 years and older	Target participants aged **40 years** and older
Up to 18 participants	Up to **16 participants**
Content	
	Reorganized order of activities
	Emphasis on work-life balance
	Updated work-related examples, content, and activities
	Addition of stress-related content/activities
	Revised communication activity
	Revised and streamlined information about nutrition
	Reduced information about falls

Bold text indicates differences across programs.

**Table 2:** Sample characteristics by intervention condition.

	Total (*n* = 181)	CDSMP (*n =* 109)	wCDSMP (*n =* 72)	Χ^2^ or t	*p*
Age (range 23 to 72)	47.90 (±10.10)	46.51 (±9.78)	49.99 (±10.29)	2.27	0.024
Sex				0.01	0.937
Male	23 (12.9%)	14 (13.1%)	9 (12.7%)		
Female	155 (87.1%)	93 (86.9%)	62 (87.3%)		
Race				1.59	0.208
Non-Hispanic White	107 (62.2%)	68 (66.0%)	39 (56.5%)		
Racial/Ethnic Minority	65 (37.8%)	35 (34.0%)	30 (43.5%)		
Education				9.61	0.142
Some high school	2 (1.1%)	0 (0.0%)	2 (2.9%)		
High school graduate or GED	14 (8.0%)	5 (4.8%)	9 (12.9%)		
Some college or technical/vocational training	46 (26.3%)	26 (24.8%)	20 (28.6%)		
Associate’s degree	25 (14.3%)	14 (13.3%)	11 (15.7%)		
Bachelor’s degree	22 (12.6%)	15 (14.3%)	7 (1.0%)		
Postgraduate work	5 (2.9%)	4 (3.8%)	1 (1.4%)		
Postgraduate degree	61 (34.9%)	41 (39.0%)	20 (28.6%)		
Chronic Conditions					
Obesity	132 (73.3%)	74 (68.5%)	58 (80.6%)	3.20	0.074
High Cholesterol	78 (45.1%)	44 (41.9%)	34 (50.0%)	1.09	0.296
High Blood Pressure	81 (44.8%)	42 (38.5%)	39 (54.2%)	4.29	0.038
Anxiety or Other Emotional/Mental Health Condition	47 (26.4%)	30 (27.5%)	17 (23.6%)	0.35	0.557
Diabetes	44 (25.1%)	18 (17.1%)	26 (37.1%)	8.93	0.003
Musculoskeletal Injury/Disorder	41 (22.7%)	23 (21.1%)	18 (25.0%)	0.38	0.540
Depression	40 (22.1%)	21 (19.3%)	19 (26.4%)	1.28	0.258
Arthritis or Other Rheumatic Disease	36 (19.9%)	21 (19.3%)	15 (20.8%)	0.07	0.796
Digestive Diseases/Conditions	35 (19.3%)	20 (18.3%)	15 (20.8%)	0.17	0.679
Asthma	12 (6.6%)	7 (6.4%)	5 (6.9%)	0.02	0.890
Cancer	6 (3.3%)	3 (2.8%)	3 (4.2%)	0.27	0.603
Heart Disease	4 (2.2%)	1 (0.9%)	3 (4.2%)	2.12	0.146
Other Physical Injuries	4 (2.2%)	2 (1.8%)	2 (2.8%)	0.18	0.673
Chronic Bronchitis, Emphysema, or Other COPD	2 (1.1%)	1 (0.9%)	1 (1.4%)	0.09	0.766
Other Lung Diseases	2 (1.1%)	2 (1.8%)	0 (0.0%)	1.34	0.248
Other Chronic Condition	25 (13.9%)	18 (16.5%)	7 (9.9%)	1.59	0.207
Number of Chronic Conditions (range 1 to 16)	3.25 (±2.02)	3.00 (±1.87)	3.64 (±2.19)	2.10	0.037
Body Mass Index (Categorical)				3.94	0.269
Normal Weight (18.5–24.9)	16 (8.9%)	13 (12.0%)	3 (4.2%)		
Overweight (25–29.9)	32 (17.8%)	21 (19.4%)	11 (15.3%)		
Obese (30–39.9)	89 (49.4%)	50 (46.3%)	39 (54.2%)		
Extremely Obese (40+)	43 (23.9%)	24 (22.2%)	19 (26.4%)		
Body Mass Index (Continuous)	34.90 (±7.91)	34.20 (±8.30)	35.95 (±7.23)	1.46	0.145
Glucose	103.84 (±36.42)	100.45 (±38.87)	108.89 (±32.03)	1.52	0.131
Elevated (>99 mg/dL)	60 (33.9%)	29 (27.4%)	31 (43.7%)	5.04	0.025
Systolic Blood Pressure	116.24 (±16.37)	114.99 (±14.83)	118.08 (±18.35)	1.40	0.217
Elevated (>119)	42 (23.6%)	24 (22.6%)	18 (25.0%)	0.13	0.716
Diastolic Blood Pressure	76.10 (±9.84)	75.98 (±8.97)	76.26 (±11.06)	0.19	0.851
Elevated (>80)	54 (30.3%)	32 (30.2%)	22 (30.6%)	0.00	0.958
Total Cholesterol	192.20 (±35.30)	190.82 (±34.94)	194.28 (±35.98)	0.64	0.524
Elevated (>199 mg/dL)	74 (41.6%)	43 (40.2%)	31 (43.7%)	0.21	0.645
LDL Cholesterol	109.95 (±30.13)	110.22 (±30.90)	109.54 (±29.13)	−0.15	0.884
Elevated (>129 mg/dL)	45 (25.4%)	30 (28.0%)	15 (21.4%)	0.98	0.323
Proportion of Sessions Attended (Categorical)				19.73	<0.001
75–100% Sessions	76 (42.0%)	51 (46.8%)	25 (34.7%)		
50–74% Sessions	56 (30.9%)	41 (37.6%)	15 (20.8%)		
25–49% Sessions	24 (13.3%)	10 (9.2%)	14 (19.4%)		
<25% Sessions	25 (13.8%)	7 (6.4%)	18 (25.0%)		
Proportion of Sessions Attended (Continuous)	0.61 (±0.28)	0.68 (±0.24)	0.52 (±0.31)	−3.80	<0.001

**Table 3:** Latent change regression analyses.

	CDSMP (Usual Care)					wCDSMP (Workplace−Tailored)					Between−Group Difference	
	Baseline Mean (SE)	*n*	*u*_Δ_ (S.E.)	*p*	Effect Size	Baseline Mean (SE)	*n*	*u*_Δ_ (S.E.)	*p*	Effect Size	*u*_Δ_ Difference (SE)	*p*
Physically Unhealthy Days	4.48 (0.78)	108	−2.13 (1.65)	0.198	−0.04	6.06 (0.73)	72	−2.07 (0.87)	0.018	−0.02	8.34 (4.48)	0.063
Mentally Unhealthy Days	6.25 (0.79)	106	2.61 (1.02)	0.010	0.05	5.33 (0.72)	72	−1.75 (0.94)	0.062	−0.04	−2.32 (3.53)	0.512
Stress	5.53 (0.32)	109	0.42 (0.45)	0.354	0.06	4.98 (0.25)	71	−0.84 (0.55)	0.127	−0.10	0.06 (1.54)	0.969
Pain	2.75 (0.31)	109	0.66 (0.50)	0.179	0.09	2.40 (0.29)	71	−0.90 (0.46)	0.052	−0.01	0.28 (1.92)	0.883
Fatigue	4.41 (0.26)	107	0.18 (0.46)	0.697	0.03	4.46 (0.25)	70	−2.88 (0.92)	0.002	−0.17	−3.68 (1.31)	0.005
Sleep Problems	3.70 (0.24)	109	−0.70 (0.54)	0.109	−0.12	3.66 (0.27)	72	−0.22 (0.56)	0.694	−0.04	0.72 (1.24)	0.560
Depression	5.40 (0.40)	109	−0.80 (1.18)	0.500	−0.06	5.58 (0.30)	72	−1.07 (0.60)	0.077	−0.07	0.63 (2.29)	0.782
Eating Behavior—Fast Food Intake (Past Week)	2.74 (0.13)	109	−0.59 (0.34)	0.082	−0.34	2.65 (0.15)	72	−0.76 (0.29)	0.009	−0.27	−1.04 (1.04)	0.317
Eating Behavior—Fruit/Vegetable Intake (Past Week)	2.76 (0.09)	109	0.56 (0.34)	0.097	0.25	2.75 (0.22)	72	0.36 (0.20)	0.077	0.17	−0.01 (1.05)	0.991
Eating Behavior—Soda/Sugar Beverage Intake (Past Week)	1.61 (0.09)	108	0.15 (0.49)	0.765	0.05	1.69 (0.17)	72	−0.78 (0.35)	0.028	−0.24	−2.70 (0.92)	0.003
Physical Activity—Days Exercise (Past Week)	1.43 (0.15)	108	−0.84 (0.51)	0.102	−0.19	1.34 (0.26)	72	0.28 (0.35)	0.424	0.07	2.88 (1.40)	0.039
Sedentary Behavior on Work Days	9.02 (0.49)	109	−0.17 (1.02)	0.870	−0.01	9.66 (0.74)	72	−4.49 (1.90)	0.018	−0.02	−14.22 (9.24)	0.124
Self-Efficacy for Managing Chronic Disease	7.70 (0.23)	86	0.33 (0.73)	0.657	0.09	7.22 (0.41)	63	−0.28 (0.72)	0.695	−0.06	−2.63 (1.38)	0.056
Prescription Medication Adherence	1.24 (0.10)	96	−0.09 (0.65)	0.895	−0.06	1.22 (0.24)	65	−0.03 (0.30)	0.923	−0.02	−1.20 (1.15)	0.297
Patient–Provider Communication	3.28 (0.12)	109	0.41 (0.43)	0.334	0.26	3.28 (0.14)	72	0.46 (0.21)	0.031	0.33	1.34 (0.83)	0.106
WLQ: Time Demands	17.06 (1.64)	105	3.12 (6.89)	0.651	0.01	23.12 (2.98)	68	1.88 (8.43)	0.824	0.00	−7.43 (21.03)	0.724
WLQ: Physical Demands	19.58 (3.80)	107	−1.06 (6.74)	0.875	0.00	21.01 (3.16)	71	3.51 (5.32)	0.525	0.00	9.94 (20.35)	0.625
WLQ: Mental Demands	16.30 (1.40)	107	1.54 (7.26)	0.832	0.00	19.80 (2.22)	71	−8.89 (4.47)	0.010	−0.02	−30.56 (14.87)	0.040
WLQ: Interpersonal Demands	7.12 (1.51)	105	−1.15 (8.82)	0.896	0.00	12.44 (2.48)	71	−3.62 (3.21)	0.529	−0.01	−9.52 (15.19)	0.531
WLQ: Output Demands	9.08 (1.07)	107	2.78 (6.06)	0.646	0.01	13.35 (2.58)	71	2.08 (4.66)	0.655	0.00	−8.57 (16.36)	0.600
Work Ability	37.55 (0.77)	109	−0.65 (1.83)	0.720	−0.01	36.63 (1.46)	72	−2.32 (1.36)	0.088	−0.04	−4.26 (7.48)	0.569
Job Stress	1.31 (0.12)	109	0.23 (0.27)	0.385	0.35	1.13 (0.17)	72	−0.18 (0.23)	0.452	−0.17	−0.35 (0.58)	0.548

Note: All analyses control for proportion of sessions attended and baseline levels of the dependent variable. Between-group difference analyses control for proportion of sessions attended, number of chronic conditions at baseline, and age. SE (standard error); WLQ (work limitations questionnaire).
